# Molecular Basis of TcdR-Dependent Promoter Activity for Toxin Production by *Clostridioides difficile* Studied by a Heterologous Reporter System

**DOI:** 10.3390/toxins15050306

**Published:** 2023-04-23

**Authors:** Xinyue Zhang, Jie Li, Chao Chen, Ya-Jun Liu, Qiu Cui, Wei Hong, Zhenghong Chen, Yingang Feng, Guzhen Cui

**Affiliations:** 1 Key Laboratory of Microbiology and Parasitology of Education Department of Guizhou & Key Laboratory of Medical Molecular Biology of Guizhou Province, Guizhou Medical University, Guiyang 550025, China; 2 CAS Key Laboratory of Biofuels, Shandong Provincial Key Laboratory of Synthetic Biology, Shandong Engineering Laboratory of Single Cell Oil, Qingdao Institute of Bioenergy and Bioprocess Technology, Chinese Academy of Sciences, Qingdao 266101, China; 3 Joint Laboratory of Helicobacter Pylori and Intestinal Microecology of Affiliated Hospital of Guizhou Medical University, Guiyang 550025, China; 4 Shandong Energy Institute, Qingdao 266101, China; 5 Qingdao New Energy Shandong Laboratory, Qingdao 266101, China; 6 University of Chinese Academy of Sciences, Beijing 100049, China

**Keywords:** *Clostridioides difficile*, enterotoxins, σ factor, heterologous system, *Bacillus subtilis*

## Abstract

The alternative σ factor TcdR controls the synthesis of two major enterotoxins: TcdA and TcdB in *Clostridioides difficile*. Four potential TcdR-dependent promoters in the pathogenicity locus of *C. difficile* showed different activities. In this study, we constructed a heterologous system in *Bacillus subtilis* to investigate the molecular basis of TcdR-dependent promoter activity. The promoters of the two major enterotoxins showed strong TcdR-dependent activity, while the two putative TcdR-dependent promoters in the upstream region of the *tcdR* gene did not show detectable activity, suggesting that the autoregulation of TcdR may need other unknown factors involved. Mutation analysis indicated that the divergent -10 region is the key determinant for different activities of the TcdR-dependent promoters. Analysis of the TcdR model predicted by AlphaFold2 suggested that TcdR should be classified into group 4, i.e., extracytoplasmic function, σ^70^ factors. The results of this study provide the molecular basis of the TcdR-dependent promoter recognition for toxin production. This study also suggests the feasibility of the heterologous system in analyzing σ factor functions and possibly in drug development targeting these factors.

## 1. Introduction

**Figure 1:**
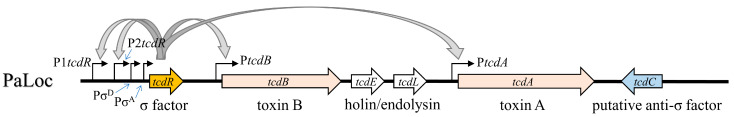
The pathogenicity locus (PaLoc) of *Clostridioides difficile*. Four TcdR-dependent promoters are located before the genes *tcdR*, *tcdA,* and *tcdB*.

The human enteropathogen *Clostridioides difficile* is an anaerobic Gram-positive spore-forming bacterium, and *C. difficile* infection (CDI) is a leading cause of worldwide antimicrobial-associated diarrhea in hospitalized elderly patients [1,2]. *Clostridioides difficile* produces two enterotoxins, TcdA and TcdB, which are the major virulence factors in CDI pathogenesis, while about 20% of *C. difficile* strains produce an additional toxin CDT whose role in the infection is less clear [3,4]. TcdA and TcdB are glucosyltransferases belonging to the large clostridial toxin (LCT) family. The genes encoding TcdA and TcdB are located in a DNA region termed the ‘pathogenicity locus’ (PaLoc); this locus contains four additional genes encoding TcdR, TcdE, TcdL, and TcdC which may play regulatory roles in toxin production and secretion (Figure 1) [4,5]. TcdA and TcdB do not contain a signal sequence for secretion, and their transport can be facilitated by the holin/endolysin system comprising TcdE and TcdL [6,7].

TcdR is a σ-factor belonging to the σ^70^ family of RNA polymerase σ-factors and it is the only positive regulator of toxin production in *C. difficile* [5,8,9]. TcdC may negatively regulate toxin expression, and it was thought to function as a membrane-bound anti-σ-factor [10], but recent studies have questioned its direct interaction with TcdR [11]. The upstream regions of both the *tcdA* and the *tcdB* genes contain a TcdR-dependent promoter, while the upstream region of the *tcdR* gene also contains two putative TcdR-dependent promoters as well as σ^D^-dependent and σ^A^-dependent promoters [12,13,14]. The PaLoc genes are further regulated by several repressors including CcpA, CodY, and RstA [5,14].

A previous study showed that TcdR can stimulate the transcription of fusion of the *tcdR* promoter region to a β-glucuronidase gene in *C. difficile* or *Clostridium perfringens* and two putative promoters P1*tcdR* and P2*tcdR* were proposed for TcdR recognition [12]. However, in a later study, P1*tcdR* and P2*tcdR* did not show detectable activity using alkaline phosphatase as a reporter in *C. difficile*, while the promoters of *tcdA* and *tcdB* showed high and low activities with about a twenty-fold difference [14]. It has been noticed that the -35 region of the four promoters is largely conserved while the -10 region is divergent [12], but the functional significance of this phenomenon is still not clear. In this study, we constructed a heterologous reporter system using β-galactosidase LacZ as the reporter and *Bacillus subtilis* as the host. Using this system, we analyzed the activities of different TcdR-dependent promoters and investigated the key determinants in the promoter for the transcriptional activity. We further analyzed the structural basis of promoter recognition using the structure of TcdR predicted by AlphaFold2. The results in this study provide the molecular basis of the TcdR-dependent promoter activity for toxin production, and the method used in this study provides a new tool for studying *C. difficile* toxin regulation.

## 2. Results

### 2.1. Construction of B. subtilis Heterologous Reporter System for Studying the Promoter Activity Controlled by TcdR

**Figure 2:**
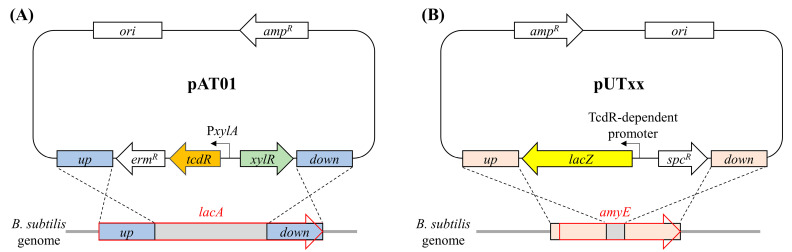
The *B. subtilis* heterologous reporter system. (**A**) The plasmid pAT01 derived from pAX01 [18] contains the xylose-induced TcdR expression cassette. The cassette can be integrated into the *lacA* locus of the *B. subtilis* genome by homologous recombination. (**B**) The plasmid pUTxx (xx is a number for different promoters) derived from pUC19 contains the promoter–reporter cassette using the β-galactosidase LacZ from *Escherichia coli* as the reporter. The cassette can be integrated into the *amyE* locus.

*B. subtilis* is a safe model bacterium with many available genetic tools. It has been widely used in protein heterologous expression and functional studies [15,16]. Since there is no ortholog of TcdR in *B. subtilis*, the bacterium should be suitable for studying promoter activity controlled by TcdR. By referring to a previous report [17], we constructed a *B. subtilis* reporter system that contains two parts (Figure 2): one is the xylose-induced TcdR expression cassette integrated into the *lacA* locus of *B. subtilis* genome; the other is the promoter-reporter cassette integrated into the *amyE* locus. The expression of TcdR is under the control of the promoter P*xylA*, which is repressed by XylR. The repression can be relieved by adding xylose to the media. The β-galactosidase LacZ from *Escherichia coli* was used as the reporter which is under the control of a TcdR-dependent promoter. After the two cassettes are integrated into the *B. subtilis* genome, the xylose-induced LacZ activity of the cell lysate could indicate the strength of the promoter recognized by TcdR.

### 2.2. Strength of Different TcdR-Dependent Promoters in the PaLoc

**Figure 3:**
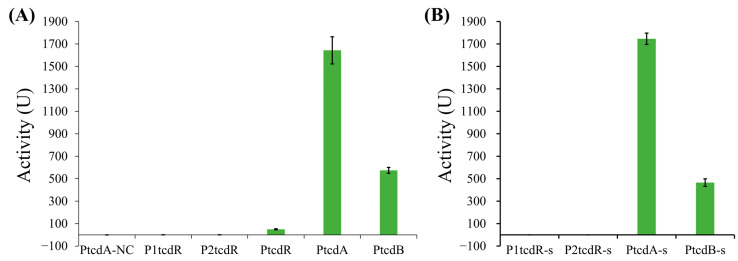
The activities of TcdR-dependent promoters. (**A**) The activities of long-version promoters. PtcdA-NC is the negative control with the integration of the promoter–reporter cassette but without the TcdR expression cassette. (**B**) The activities of short-version promoters which contain only the region from the -35 element to the -10 element.

According to previous reports [8,12,14,19], the PaLoc of *C. difficile* contains four TcdR-dependent promoters (Figure 1, Table S1). P1*tcdR* and P2*tcdR* are located before the gene of the σ factor TcdR, while P*tcdA* and P*tcdB* are located before the genes of the two toxins TcdA and TcdB, respectively. We used the *B. subtilis* reporter system to detect the activities of these promoters (Figure 3A and Figure S1). The results showed that the promoter of *tcdA* has the strongest activity and that the promoter of *tcdB* also has strong activity. In contrast, the two putative promoters of *tcdR* did not show detectable activity, while a longer region containing both the TcdR-dependent promoters and the σ^D^-dependent promoter Pσ^D^ showed weak but significant activity. These results are consistent with the data in the literature. For example, the mRNA level of *tcdA* is about two-fold of the *tcdB* mRNA level in the early stationary phase in *C. difficile* [19]. A later study using alkaline phosphatase as a reporter in *C. difficile* showed that the activity of P*tcdA* is about twenty-fold higher than P*tcdB*, while P1*tcdR* and P2*tcdR* did not show a significant difference compared to the promoter-less reporter. Moreover, the study also showed P*tcdR,* that contained the Pσ^D^, had a weak but detectable activity [14]. The consistent results between our work and previous studies indicated that the *Bacillus* heterologous system constructed in this work is effective for the study of TcdR-dependent promoters.

Considering that the promoter regions may contain regulatory elements affecting the activity [14], we shortened these promoters to only contain the region from the -35 element to the -10 element. Activity measurements showed similar activities of these short promoters with the long promoters (Figure 3B), suggesting that the removed regions were negligible in the *Bacillus* system. These results indicated that the four TcdR-dependent promoters have different activities, and this difference should be attributed to the intrinsic -35 and -10 regions of these promoters.

### 2.3. The -10 Elements Determine the Activity Difference of TcdR-Dependent Promoters

**Figure 4:**
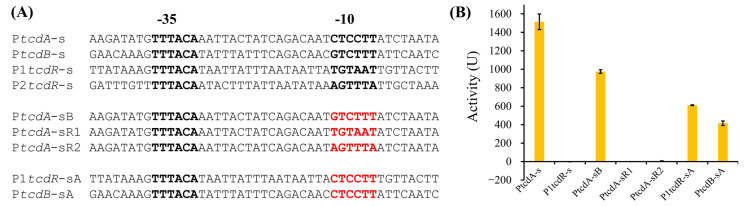
The -10 regions determine the activities of different TcdR-dependent promoters. (**A**) The alignment of TcdR-dependent promoters. The -35 and -10 elements are indicated in bold font and the mutations are in red. (**B**) The activities of -10 region-exchanged promoters.

To investigate the reason for the different activities of the four TcdR-dependent promoters, we performed sequence alignment for the four promoters (Figure 4A). Similar to the previous observation [12], the alignment showed that the -35 elements of the four promoters are completely conserved, but the -10 elements are poorly conserved. Moreover, the -10 elements of P*tcdA* and P*tcdB* are similar (four of six bases are identical), while the -10 elements of P1*tcdR* and P2*tcdR* are more different from P*tcdA* and P*tcdB*. Therefore, we suspected that the -10 element is the key to determining the activity of different promoters. We constructed several mutant promoters by exchanging the -10 regions (6 bp) of the four TcdR-dependent promoters. As expected, the P*tcdA*-sR1 and P*tcdA*-sR2 showed no activity in the reporter system, while P1*tcdR*-sA showed high activity (about 30% activity of wild-type P*tcdA*-s) (Figure 4B). This result demonstrated that the -10 region is the key determinant for the different activities of various TcdR-dependent promoters. The P*tcdA*-sB showed lower activity than P*tcdA*-s but still higher than P*tcdB*-s, while P*tcdB*-sA showed similar activity as P*tcdB*-s. These results, as well as the lower activity of P1*tcdR*-sA in comparison with the activity of wild-type P*tcdA*-s, suggested that some other positions outside the -35 and -10 elements might also modulate the promoter activity.

### 2.4. Key Nucleotide Residues in the -10 Elements of PtcdA and P1tcdR

**Figure 5:**
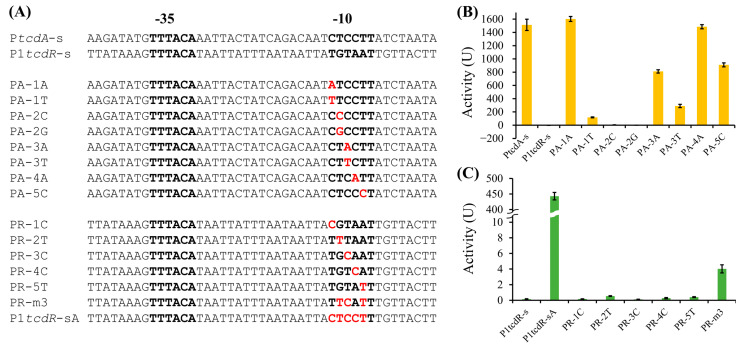
Mutation analysis in the -10 region. (**A**) The sequences of the promoter mutants. The -35 and -10 elements are indicated in bold font. The mutated sites are shown in red. (**B**) The activities of the P*tcdA*-s mutants. (**C**) The activities of the P1*tcdR*-s mutants.

To further elucidate the key nucleotide residues in the -10 element of the TcdR-dependent promoter, we mutated the -10 elements of P*tcdA*-s and P1*tcdR*-s (Figure 5). A single mutation in the first position (C→A) or the fourth position (C→A) of the -10 element of P*tcdA*-s did not alter the activity of the promoter, while other mutations significantly reduced the activity of the promoter (Figure 5B). Particularly, the two mutations at the second position resulted in a complete loss of activity. These results indicate that the nucleotides at the second, third, and fifth positions play more significant roles in TcdR-dependent promoter activity. 

We then mutated each position of the -10 element in P1*tcdR*-s to the same nucleotide in P*tcdA*-s and checked if the activity of P1*tcdR*-s could be restored (Figure 5C). Among the five single-nucleotide mutations, only the mutant at the second position (G→T) showed very weak activity. Furthermore, the triple mutation at the second, third, and fifth positions could restore low but significant activity. Therefore, the promoter activity should depend on multiple nucleotides in the -10 element, and the second position is more crucial than other positions.

### 2.5. Structural Basis of TcdR-Dependent Promoter Recognition

**Figure 6:**
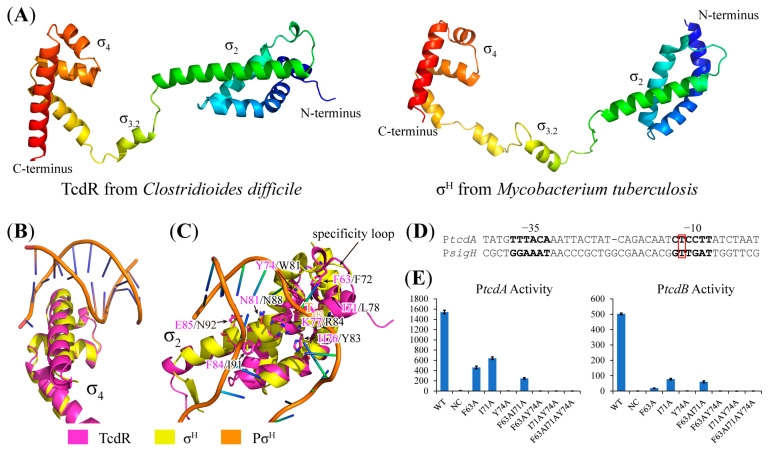
The structure model of TcdR represents structural features consistent with the ECF-type σ factor. (**A**) The structure model of TcdR predicted by AlphaFold2 and the reported structure of the ECF-type σ factor σ^H^ in RNA polymerase transcription initiation complex (TIC) from *Mycobacterium tuberculosis* (PDB 5ZX2). Both structures from the N-terminus to the C-terminus are colored in a rainbow from blue to red. (**B**) Superimposition of the σ4 domain structures of TcdR (green) and σ^H^ (yellow). The promoter DNA (orange) bound by σ^H^ is also shown. (**C**) Superimposition of the σ2 domain structures of TcdR (green) and σ^H^ (yellow). Some key residues for -10 element recognition are shown as sticks. (**D**) Alignment of the TcdR-dependent and σ^H^-dependent promoters. The -35 and -10 elements are indicated in bold font. The position of T_-12_ is indicated by a red rectangle. (**E**) The activities of the P*tcdA* and P*tcdB* with TcdR or its mutants. WT, wild-type TcdR; NC, negative control, which has no TcdR expression cassette. The structure figures were prepared using the PyMol software (Schrödinger).

TcdR showed very low homology with known group 1–4 σ^70^ factors and thus TcdR was classified into group 5 [9]. Currently, there is no experimental structure of TcdR available. To obtain structural insight into the TcdR-dependent promoter recognition, we analyzed the TcdR structure model predicted by AlphaFold2 [20]. Compared with the structure of group 4 (also called extracytoplasmic function (ECF)) σ factor σ^H^ in the RNA polymerase transcription initiation complex (TIC) from *Mycobacterium tuberculosis* (PDB 5ZX2) [21], the TcdR structure model resembles ECF σ factors that consist of two domains (σ2 and σ4) linked by a linker containing a short helix (σ3.2) despite the low sequence homology (Figure 6A). The σ4 domains of both TcdR and σ^H^ contain a helix-turn-helix motif which could bind to the major groove of -35 promoter DNA (Figure 6B). The surface residues of the motif are different in the two σ4 domains, implying specific −35 element recognition by TcdR. The σ2 domain of TcdR has a long helix containing conserved residues (N81, K77, and F84) for DNA unwinding similar to ECF σ factors (Figure 6C). The specific loop of ECF σ factors recognizes a T base in the -10 element by W81, F72, and L78 in σ^H^, and the corresponding region of TcdR contains similar residues (Y74, F63, and I71), which may be proposed to recognize the crucial T base at the second position of the -10 region of the TcdR-dependent promoter because the same T bases exist in the alignment of the TcdR- and σ^H^-dependent promoters (Figure 6D). The role of these residues was further confirmed by the assays using TcdR mutants containing single or combined mutations (Figure 6E). Single or combined mutations of F63A and I71A significantly reduced the activities of both P*tcdA* and P*tcdB*, while the mutation of Y74A or any combined mutation containing Y74A abolished the activities of the promoters, indicating that Y74 plays more crucial roles in promoter recognition. The short helix (σ3.2) is the key part in the active site cleft of the RNAP core enzyme. All these features are consistent with the structural features of ECF σ factors, suggesting that TcdR is also an ECF σ factor, i.e., a group 4 σ^70^ factor.

## 3. Discussion

TcdR controls the expression of the major toxins in *C. difficile* by recognizing the promoters of their genes [8]. TcdR was also thought to be auto-regulated by recognizing two putative promoters in the upstream region of the *tcdR* gene, but a later study showed no detectable activity of these putative promoters [12,14]. This study, using the *Bacillus* heterologous system, confirmed that the two promoters do not have TcdR-dependent activity. The reason, elucidated by mutation analysis, is the -10 region in these promoters.

TcdR and homologous σ factors in several pathogens for toxin regulation were considered to be a new group (group 5) of σ^70^ factors because of the low sequence identity compared with group 1–4 σ^70^ factors [9,22]. However, the four groups of σ^70^ factors are mainly classified according to the domain organization [23,24]. Group 1 is the housekeeping σ factors containing four domains conserved in bacteria. Group 2–4 σ^70^ factors are alternative σ factors with fewer domains. The group 4 σ factors (also called ECF σ factors) are most functionally divergent but with the simplest domain organization containing only the σ2 and σ4 domains by a linker (σ3.2). Our analysis in this study showed that the structure, domain organization, and promoter recognition of TcdR are consistent with ECF σ factors, suggesting that it should be classified into ECF σ factors. A previous study showed that the expression of TcdR is controlled by temperature [25], conforming to the environmental sensing function of ECF σ factors. Further study on the structure of TcdR in the complex with RNAP and promoters may clarify the classification and structural basis of promoter recognition.

The *Bacillus* heterologous system constructed in this study has several advantages in the study of the TcdR function. The genetic manipulation of *Bacillus subtilis* as a safe model microorganism is more convenient than *C. difficile*. This heterologous system can avoid the manipulation of pathogens; therefore, it can be easily operated in more complex and large-scale equipment. *B. subtilis* neither contains TcdR nor enterotoxins; therefore, the interference of other factors in the original host in the results is reduced. A similar heterologous system has been constructed to study a distinct σ factor σ^I^ which regulates the expression of a multienzyme complex, cellulosome, for lignocellulose degradation [17,26,27,28]. Our study in this paper indicated that the *Bacillus* heterologous system can be used in the study for more σ factors specific in pathogens and other bacteria that are difficult to genetically manipulate. For example, besides TcdR, *C. difficile* contains distinct σ factors CsfT and CsfU, which also play important roles in the pathogenesis [29,30]. The function and regulation of these factors could also be studied using the *Bacillus* system. Furthermore, RNAP and σ factors are also important targets for drug development [31,32]. The *Bacillus* system could be used to screen inhibitors more conveniently with high-throughput equipment. It should be noted that the heterologous system may also have disadvantages in these potential studies and applications. For example, the studied promoters may contain binding sites for some *Bacillus*-specific transcription factors which may cause results not relevant to *C. difficile*. Careful setup of control experiments should be conducted when using the heterologous system. Ultimately, important findings from the heterologous system should be assessed in the native organism.

## 4. Conclusions

In conclusion, this study constructed a *Bacillus* heterologous system to study the TcdR-dependent promoter activity of TcdR for toxin expression in *C. difficile*, and the results revealed the molecular basis of the different promoter activities. Structural analysis suggested that TcdR has a domain organization and promoter recognition mechanism consistent with ECF σ factors and thus it should be classified into the ECF σ factors. The heterologous system is valuable in future studies for σ factor functions and drug development targeting these factors.

## 5. Materials and Methods

### 5.1. Bacterial Strains, Growth Medium, and Culture Conditions

The bacterial strains used in this study are listed in Table S2. *Escherichia coli* and *B. subtilis* were routinely grown in LB media at 37 °C. SM1 and SM2 media were used for the transformation of *B. subtilis* [33]. MCSE media were used for the β-galactosidase activity assay [34]. When necessary, 100 μg/mL ampicillin, 4 μg/mL erythromycin, and 100 μg/mL spectinomycin were supplemented to the media.

The plasmids and primers used in this study are listed in Tables S3 and S4. All the plasmids were constructed using the One Step Cloning Kit (Vazyme Biotech Co., Ltd., Nanjing, China) based on the in-fusion technique.

Plasmid pAT01 was constructed from the vector pAX01 [18,35] to express TcdR (GenBank: CAJ67491) of *C. difficile* in *B. subtilis*. The *tcdR* gene was amplified from the genomic DNA of *C. difficile* 630 by PCR using the primers T01 and T02. The PCR product was ligated with the linearized pAX01 vector, which was prepared by PCR using primers T03 and T04, forming the plasmid pAT01. The plasmid pAT01 contains the *tcdR* gene controlled by the xylose-inducible promoter P*xylA* and can integrate the TcdR expression cassette and the erythromycin resistance gene into the *B. subtilis lacA* chromosomal locus by homologous recombination. The plasmids for the expression of TcdR mutants were constructed by the QuikChange mutagenesis method using pAT01 as the template.

A series of plasmids of pUT were constructed from the vector pUC19 and each contains a TcdR-dependent promoter and the β-galactosidase *lacZ* from *E. coli* as a reporter gene, a spectinomycin resistance gene for screening, and homologous regions for integration in the *amyE* locus of *B. subtilis*. The promoter fragments of P*tcdA*, P*tcdB*, P1*tcdR*, P2*tcdR*, P*tcdR*, and P2*tcdR*-s were amplified from the genomic DNA of *C. difficile* 630 by PCR using the primers U01-F/R, U03-F/R, U05-F/R, U07-F/R, U11-F/R, and U16-F/R, respectively, and the pUC19 vector was linearized using the corresponding primers U02-F/R, U04-F/R, U06-F/R, U08-F/R, U12-F/R, and U17-F/R, respectively. The PCR products of the promoters, the *lacZ* gene, the spectinomycin resistance gene, and the homologous regions of *amyE* were ligated to the linearized pUC19 vectors, obtaining the plasmids pUT04, pUT07, pUT08, pUT09, pUT11, and pUT15. The reporter plasmids pUT12, pUT13, and pUT14 for the short-version promoters P*tcdA*-s, P*tcdB*-s, and P1*tcdR*-s were constructed by the deletion of the 5′ and 3′ regions of the long-version promoters using two rounds of PCR for the plasmid. The first round of PCR was performed using the primers U13-F/R, U14-F/U13-R, and U15-F/U13-R with the plasmids pUT04, pUT07, and pUT08, respectively, as the template. The PCR products were directly transformed into *E. coli* Top10 and were cyclized in the cells. The second round of PCR was performed using the primers U13-2-F/R, U14-2-F/R, and U15-2-F/R with the plasmids obtained in the first round as the template. The second-round PCR products were transformed into *E. coli* Top10, obtaining the plasmids pUT12, pUT13, and pUT14. To investigate the key regions and nucleotides recognized by TcdR, the pUT plasmids containing different length regions and mutants of TcdR-dependent promoters were constructed by PCR or the QuikChange mutagenesis method.

### 5.2. Construction of B. subtilis Strains

The natural competence method was used for the transformation of *B. subtilis* as the reported protocol [33]. After the culture and screening of plates containing appropriate antibiotics, the transformants were confirmed by colony PCR. Two rounds of transformation were performed to obtain each mutant strain (Table S2). For the study of TcdR-dependent promoters, the plasmid pAT01 was first transformed into *B. subtilis* 168, obtaining the strain 168R01 after screening the double-crossover in the *lacA* locus. Then, each of the pUT series plasmids was transformed into TR01, obtaining the final strain (168AR01, 168BR01, and 168RR01-168RR04) after screening the double-crossover in the *amyE* locus for reporting the activity of the TcdR-dependent promoter. For the study of TcdR mutants, each of the plasmids pUT04 and pUT07 containing the reporter cassette of P*tcdA* and P*tcdB*, respectively, was first transformed into the *B. subtilis* 168, and then the plasmids containing the expression cassette of TcdR mutant were transformed into each of these strains.

### 5.3. β-Galactosidase Activity Assay

The measurement of the β-galactosidase activity was performed as previously described with minor modifications [17]. The *B. subtilis* mutant strain was inoculated and cultivated in the MCSE medium, with shaking and 250 rpm at 37 °C, until the optical density at 600 nm (OD_600nm_) reached 0.4–0.5. Briefly, 1% xylose was supplemented to induce the expression of the downstream gene under the control of the promoter P*xylA* for 3 h at 37 °C. Then, 3 mL of the bacterial cells were collected by centrifugation at 15,493× *g* for 10 min. The cell pellets were washed twice with Z-buffer (60 mM Na_2_HPO_4_·2H_2_O, 40 mM NaH_2_PO_4_·H_2_O, 10 mM KCl, 1 mM MgSO_4_·7H_2_O, pH 7.0) and resuspended with 600 μL of the working buffer (the Z-buffer with additional 20 mM β-mercaptoethanol). In total, 50 μL of suspension was used to determine the value of OD_600nm_, and the rest was lysed by ultrasonication. The lysate was centrifugated at 15,493× *g* for 10 min. The reaction was initiated by adding 10 μL of ortho-nitrophenyl-β-galactoside (ONPG, with the final concentration of 1.31 mg/mL) into 90 μL of the supernatant, and the mixture was incubated at 37 °C for 4–60 min. Then, the reaction was terminated by adding 200 μL of 1 M Na_2_CO_3_ to 40 μL of the sample. The released 2-nitrophenol (ONP) was measured by determining the absorbance at 420 nm (A_420nm_). All the β-galactosidase activities were normalized with the OD_600nm_, and one unit of enzyme activity was defined as the amount of β-galactosidase that releases 1 nmol of ONP per minute.

## Data Availability

The data presented in this study are available in this article and supplementary materials.

## Supplementary Materials

The following supporting information can be downloaded at https://www.mdpi.com/article/10.3390/toxins15050306/s1: Figure S1: The activities of long version TcdR-dependent promoters with and without the xylose induction; Table S1: Promoter sequences used in this study; Table S2: Bacterial strains and plasmids used in this study; Table S3: Plasmids used in this study; Table S4: Primers used in this study.
