# Supplementary material for: Molecular Basis of TcdR-Dependent Promoter Activity for Toxin Production by Clostridioides difficile Studied by a Heterologous Reporter System
Source: Toxins (Basel). 2023 Apr 23;15(5):306. doi: 10.3390/toxins15050306 (PMC10223161; doi:10.3390/toxins15050306)
Supplement: Supplementary file 1 [file toxins-15-00306-s001.zip › toxins-2267708-supplementary.pdf]

Supplementary Materials: Molecular Basis of TcdR-Dependent Promoter Activity for Toxin Production by *Clostridioides difficile* Studied by a Heterologous Reporter System

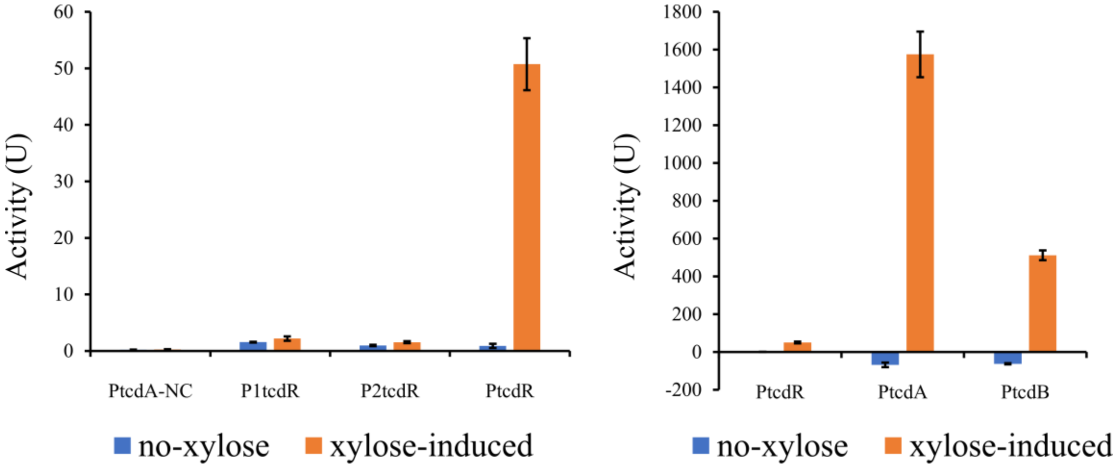

Figure S1. The activities of long-version TcdR-dependent promoters with and without the xylose induction. The activities shown in Figure 3A are the difference between the xylose-induced activity and the no-xylose activity.

Table S1. Promoter sequences used in this study. The shorter version promoters are underlined and the -35 and -10 elements are indicated in bold. The σ^D-dependent promoter is underlined with a dashed line.

Promoter	Sequence
P*tcdA*	CACAAAGATGGTGCATGGTCAGTTGGTAAAATCTATTAAGCTACATTAGTTACAGATATC<br>ACAAACTATAATAGTTAAACATAGAAATATGTGTAAATTGTGATGGAAATTATTCAAAAA<br>CACAAAAATACGTGATGAAGGACAAAATGATATAGAAAATAAGTATCAAACCTTAATAA<br>ATGATTTAATTGATAGTTTAAAAGTTATAGGAAAAATATATAAAGAAATAAAAACATTAA<br>AAAAATATAAGATATG**TTTACA**AATTACTATCAGACAAT**CTCCTT**ATCTAATAGAAGAG
P*tcdB*	TTAATGAGCTTAAAGAAATATTTACAATAGAAATCAAATTTTAGAATTAACTTTATTGTAA<br>AATCAATAACTTAATCTAAGAATATCTTAATTTTTATATTTTATATAGAACAAAG**TTTACA**<br>TATTTATTTCAGACAAC**GTCTTT**ATTCAATCGAAGAGC
P1*tcdR*	AACAAAATATTAAATAATTCTACTCTGATAGTTTGTTAAAAAAATAATAAAAAATATTAA<br>TAAACAAAAAAAAAATTATCTTAAGAGAGGAGAATGTTCTAAAATATAAAAAGGTTTCT<br>AGATTTCATAAAAGATACTATTTTAGTCTTGAAAATATTTAGTTTGAAAAGATTTTAATTT<br>AATGATTGATTAAGTTAAAAATGTGTATGTAATAAATTTTATTTTATTTATTGACTAAATTA<br>TAAAG**TTTACA**TAATTATTTAATAATTA**TGTAAT**TGTTACTTGAAAATTGATCTATTTTAA<br>AA
P2*tcdR*	ATTTTAAAATCTAGTTATAACTTCAAAAAAGACTGAAAATTAAGAAAAAAGAAATATAA<br>ATATAAAAATATGTCATATAGATTTTTTTTATTTTACTTTAATAAAATGATTTGTT**TTTACA**<br>ATACTTTATTAATATAA**AGTTTA**TTGCTAAAATACTTTATTTA
P*tcdR*	AACAAAATATTAAATAATTCTACTCTGATAGTTTGTTAAAAAAATAATAAAAAATATTAA<br>TAAACAAAAAAAAAATTATCTTAAGAGAGGAGAATGTTCTAAAATATAAAAAGGTTTCT<br>AGATTTCATAAAAGATACTATTTTAGTCTTGAAAATATTTAGTTTGAAAAGATTTTAATTT<br>AATGATTGATTAAGTTAAAAATGTGTATGTAATAAATTTTATTTTATTTATTGACTAAATTA<br>TAAAG**TTTACA**TAATTATTTAATAATTA**TGTAAT**TGTTACTTGAAAATTGATCTATTTTAA<br>AATCTAGTTATAACTTCAAAAAAGACTGAAAATTAAGAAAAAAGAAATATAAATATAAA<br>AATATGTCATATAGATTTTTTTTATTTTACTTTAATAAAATGATTTGTT**TTTACA**ATACTTTA

	TTAATATAA**AGTTTA**TTGCTAAAATACTTTATTTATTAGAAAAAGATTACTAATTAATTAT<br>TAAAATTAATGTATTCATAATGCATATTTTCATA**TAAA**ATTTAATTTATTT**GCCGATTA**TAT<br>AATTATAATGACTGATTTAATTCCAATGTTGTCAAAATTTTCAAATAAATCATCATTA

Table S2. Bacterial strains and plasmids used in this study.

Strains	Description	Source
Escherichia coli		
TOP10	For plasmid construction.	Transgen Biotech
Bacillus subtilis		
168	Wild-type host strain	Lab stock
168R01	168 *lacA*::(P*xyl-tcdR*, Erm^R).<br>The intrinsic *lacA* of 168 was replaced with the xylose-induced expression cassette of *tcdR*.	All strains below were constructed in this study
168A01	168 *amyE*::(P*tcdA-lacZ*, Spc^R).<br>The *amyE* of 168 was replaced with the LacZ reporter cassette containing the promoter of *tcdA* (P*tcdA*).	
168AR01	168 *lacA*::(P*xyl-tcdR*, Erm^R), *amyE*::(P*tcdA-lacZ*, Spc^R).<br>The *amyE* of 168R01 was replaced with the LacZ reporter cassette containing the promoter of *tcdA* (P*tcdA*).	
168BR01	168 *lacA*::(P*xyl-tcdR*, Erm^R), *amyE*::(P*tcdB-lacZ*, Spc^R).<br>The *amyE* of 168R01 was replaced with the LacZ reporter cassette containing the promoter of *tcdB* (P*tcdB*).	
168RR01	168 *lacA*::(P*xyl-tcdR*, Erm^R), *amyE*::(P1*tcdR-lacZ*, Spc^R).<br>The *amyE* of 168R01 was replaced with the LacZ reporter cassette containing the first promoter of *tcdR* (P1*tcdR*).	
168RR02	168 *lacA*::(P*xyl-tcdR*, Erm^R), *amyE*::(P2*tcdR-lacZ*, Spc^R).<br>The *amyE* of 168R01 was replaced with the LacZ reporter cassette containing the second promoter of *tcdR* (P2*tcdR*).	
168RR04	168 *lacA*::(P*xyl-tcdR*, Erm^R), *amyE*::(P*tcdR-lacZ*, Spc^R).<br>The *amyE* of 168R01 was replaced with the LacZ reporter cassette containing the long promoter region of *tcdR* (P1*tcdR* + P2*tcdR* + Pσ^D).	
168AR02	168 *lacA*::(P*xyl-tcdR*, Erm^R), *amyE*::(P*tcdA*-s-*lacZ*, Spc^R).<br>The *amyE* of 168R01 was replaced with the LacZ reporter cassette containing the short version promoter of *tcdA* (P*tcdA*-s).	
168BR02	168 *lacA*::(Pxyl-tcdR, Erm^R), *amyE*::(P*tcdB*-s-*lacZ*, Spc^R).<br>The *amyE* of 168R01 was replaced with the LacZ reporter cassette containing the short version promoter of *tcdB* (P*tcdB*-s).	
168RR05	168 *lacA*::(P*xyl-tcdR*, Erm^R), *amyE*::(P1*tcdR*-s-*lacZ*, Spc^R).<br>The *amyE* of 168R01 was replaced with the LacZ reporter cassette containing a short version of promoter P1*tcdR* (P1*tcdR*-s).	
168RR06	168 *lacA*::(P*xyl-tcdR*, Erm^R), *amyE*::(P2*tcdR*-s-*lacZ*, Spc^R).<br>The *amyE* of 168R01 was replaced with the LacZ reporter cassette containing a short version of promoter P2*tcdR* (P2*tcdR*-s).	

168AR03	168 *lacA*::(P*xyl*-*tcdR*, Erm^R), *amyE*::(P*tcdA*-sR1-*lacZ*, Spc^R).<br>The *amyE* of 168R01 was replaced with the LacZ reporter cassette containing a mutant promoter (P*tcdA*-sR1) in which the -10 element of promoter P*tcdA*-s was replaced with the corresponding sequence in promoter P1*tcdR*-s.
168BR03	168 *lacA*::(P*xyl*-*tcdR*, Erm^R), *amyE*::(P*tcdB*-sA-*lacZ*, Spc^R).<br>The *amyE* of 168R01 was replaced with the LacZ reporter cassette containing a mutant promoter (P*tcdB*-sA) in which the -10 element of promoter P*tcdB*-s was replaced with the corresponding sequence in promoter P*tcdA*-s.
168RR07	168 *lacA*::(P*xyl*-*tcdR*, Erm^R), *amyE*::(P1*tcdR*-sA-*lacZ*, Spc^R).<br>The *amyE* of 168R01 was replaced with the LacZ reporter cassette containing a mutant promoter (P1*tcdR*-sA) in which the -10 element of promoter P1*tcdR*-s was replaced with the corresponding sequence in promoter P*tcdA*-s.
168AR04	168 *lacA*::(P*xyl*-*tcdR*, Erm^R), *amyE*::($P_{A\text{-}1A}$-*lacZ*, Spc^R).<br>The *amyE* of 168R01 was replaced with the LacZ reporter cassette containing the mutant promoter ($P_{A\text{-}1A}$) in which the first position of the -10 element of P*tcdA*-s was mutated to base A.
168AR05	168 *lacA*::(P*xyl*-*tcdR*, Erm^R), *amyE*::($P_{A\text{-}1T}$-*lacZ*, Spc^R).<br>The *amyE* of 168R01 was replaced with the LacZ reporter cassette containing the mutant promoter $P_{A\text{-}1T}$ in which the first position of the -10 element of P*tcdA*-s was mutated to base T.
168AR06	168 *lacA*::(P*xyl*-*tcdR*, Erm^R), *amyE*::($P_{A\text{-}2C}$-*lacZ*, Spc^R).<br>The *amyE* of 168R01 was replaced with the LacZ reporter cassette containing the promoter $P_{A\text{-}2C}$ in which the second position of the -10 element of P*tcdA*-s was mutated to base C.
168AR07	168 *lacA*::(P*xyl*-*tcdR*, Erm^R), *amyE*::($P_{A\text{-}2G}$-*lacZ*, Spc^R).<br>The *amyE* of 168R01 was replaced with the LacZ reporter cassette containing the promoter $P_{A\text{-}2G}$ in which the second position of the -10 element of P*tcdA*-s was mutated to base G.
168AR08	168 *lacA*::(P*xyl*-*tcdR*, Erm^R), *amyE*::($P_{A\text{-}3A}$-*lacZ*, Spc^R).<br>The *amyE* of 168R01 was replaced with the LacZ reporter cassette containing the promoter $P_{A\text{-}3A}$ in which the third position of the -10 element of P*tcdA*-s was mutated to base A.
168AR09	168 *lacA*::(P*xyl*-*tcdR*, Erm^R), *amyE*::($P_{A\text{-}3T}$-*lacZ*, Spc^R).<br>The *amyE* of 168R01 was replaced with the LacZ reporter cassette containing the promoter $P_{A\text{-}3T}$ in which the third position of the -10 element of P*tcdA*-s was mutated to base T.
168AR10	168 *lacA*::(P*xyl*-*tcdR*, Erm^R), *amyE*::($P_{A\text{-}4A}$-*lacZ*, Spc^R).<br>The *amyE* of 168R01 was replaced with the LacZ reporter cassette containing the promoter $P_{A\text{-}4A}$ in which the fourth position of the -10 element of P*tcdA*-s was mutated to base A.

168AR11	168 *lacA*::(P*xyl-tcdR*, Erm^{R}), *amyE*::($P_{A\text{-}5C}$-*lacZ*, Spc^{R}).<br>The *amyE* of 168R01 was replaced with the LacZ reporter cassette containing the promoter $P_{A\text{-}5C}$ in which the fifth position of the -10 element of P*tcdA*-s was mutated to base C.
168AR12	168 *lacA*::(P*xyl-tcdR*, Erm^{R}), *amyE*::(P*tcdA*-sB-*lacZ*, Spc^{R}).<br>The *amyE* of 168R01 was replaced with the LacZ reporter cassette containing a mutant promoter (P*tcdA*-sB) in which the -10 element of promoter P*tcdA*-s was replaced with the corresponding sequence in promoter P*tcdB*-s.
168AR13	168 *lacA*::(P*xyl-tcdR*, Erm^{R}), *amyE*::(P*tcdA*-sR2-*lacZ*, Spc^{R}).<br>The *amyE* of 168R01 was replaced with the LacZ reporter cassette containing a mutant promoter (P*tcdA*-sR2) in which the -10 element of promoter P*tcdA*-s was replaced with the corresponding sequence in promoter P2*tcdR*-s.
168RR08	168 *lacA*::(P*xyl-tcdR*, Erm^{R}), *amyE*::($P_{R\text{-}1C}$-*lacZ*, Spc^{R}).<br>The amyE of 168R01 was replaced with the LacZ reporter cassette containing the promoter $P_{R\text{-}1C}$ in which the first position of the -10 element of P1*tcdR*-s was mutated to base C.
168RR09	168 *lacA*::(P*xyl-tcdR*, Erm^{R}), *amyE*::($P_{R\text{-}2T}$-*lacZ*, Spc^{R}).<br>The *amyE* of 168R01 was replaced with the LacZ reporter cassette containing the promoter $P_{R\text{-}2T}$ in which the second position of the -10 element of P1*tcdR*-s was mutated to base T.
168RR10	168 *lacA*::(P*xyl-tcdR*, Erm^{R}), *amyE*::($P_{R\text{-}3C}$-*lacZ*, Spc^{R}).<br>The *amyE* of 168R01 was replaced with the LacZ reporter cassette containing the promoter $P_{R\text{-}3C}$ in which the third position of the -10 element of the promoter P1*tcdR*-s is mutated to base C.
168RR11	168 *lacA*::(P*xyl-tcdR*, Erm^{R}), *amyE*::($P_{R\text{-}4C}$-*lacZ*, Spc^{R}).<br>The *amyE* of 168R01 was replaced with the LacZ reporter cassette containing the promoter $P_{R\text{-}4C}$ in which the fourth position of the -10 element of P1*tcdR*-s was mutated to base C.
168RR12	168 *lacA*::(P*xyl-tcdR*, Erm^{R}), *amyE*::($P_{R\text{-}5T}$-*lacZ*, Spc^{R}).<br>The *amyE* of 168R01 was replaced with the LacZ reporter cassette containing the promoter $P_{R\text{-}5T}$ in which the fifth position of -10 element of P1*tcdR*-s was mutated to base T.
168RR13	168 *lacA*::(P*xyl-tcdR*, Erm^{R}), *amyE*::($P_{R\text{-}m3}$-*lacZ*, Spc^{R}).<br>The *amyE* of 168R01 was replaced with the LacZ reporter cassette containing the promoter $P_{R\text{-}m3}$ in which the second, third, and fifth position of the -10 element of P1*tcdR*-s was mutated to base T, C, and T, respectively.
168A02	168 *amyE*::(P*tcdA-s-lacZ*, Spc^{R}).<br>The *amyE* of 168 was replaced with the LacZ reporter cassette containing the short version promoter of *tcdA* (P*tcdA-s*).
168B02	168 *amyE*::(P*tcdB-s-lacZ*, Spc^{R}).

	The *amyE* of 168 was replaced with the LacZ reporter cassette containing the short version promoter of *tcdB* (P*tcdB-s*).
168AR02-F63A	168 *lacA*::(P*xyl-tcdR-F63A*, Erm^{R}), *amyE*::(P*tcdA-s-lacZ*, Spc^{R}). The *lacA* of 168A02 was replaced with the xylose-induced expression cassette of *tcdR-F63A*.
168AR02-I71A	168 *lacA*::(P*xyl-tcdR-I71A*, Erm^{R}), *amyE*::(P*tcdA-s-lacZ*, Spc^{R}). The *lacA* of 168A02 was replaced with the xylose-induced expression cassette of *tcdR-I71A*.
168AR02-Y74A	168 *lacA*::(P*xyl-tcdR-Y74A*, Erm^{R}), *amyE*::(P*tcdA-s-lacZ*, Spc^{R}). The *lacA* of 168A02 was replaced with the xylose-induced expression cassette of *tcdR-Y74A*.
168AR02-F63AI71A	168 *lacA*::(P*xyl-tcdR-F63AI71A*, Erm^{R}), *amyE*::(P*tcdA-s-lacZ*, Spc^{R}). The *lacA* of 168A02 was replaced with the xylose-induced expression cassette of *tcdR-F63AI71A*.
168AR02-I71AY74A	168 *lacA*::(P*xyl-tcdR-I71AY74A*, Erm^{R}), *amyE*::(P*tcdA-s-lacZ*, Spc^{R}). The *lacA* of 168A02 was replaced with the xylose-induced expression cassette of *tcdR-I71AY74A*.
168AR02-F63AY74A	168 *lacA*::(P*xyl-tcdR-F63AY74A*, Erm^{R}), *amyE*::(P*tcdA-s-lacZ*, Spc^{R}). The *lacA* of 168A02 was replaced with the xylose-induced expression cassette of *tcdR-F63AY74A*.
168AR02-F63AI71AY74A	168 *lacA*::(P*xyl-tcdR-F63AI71AY74A*, Erm^{R}), *amyE*::(P*tcdA-s-lacZ*, Spc^{R}). The *lacA* of 168A02 was replaced with the xylose-induced expression cassette of *tcdR-F63AI71AY74A*.
168BR02-F63A	168 *lacA*::(P*xyl-tcdR-F63A*, Erm^{R}), *amyE*::(P*tcdB-s-lacZ*, Spc^{R}). The *lacA* of 168B02 was replaced with the xylose-induced expression cassette of *tcdR-F63A*.
168BR02-I71A	168 *lacA*::(P*xyl-tcdR-I71A*, Erm^{R}), *amyE*::(P*tcdB-s-lacZ*, Spc^{R}). The *lacA* of 168B02 was replaced with the xylose-induced expression cassette of *tcdR-I71A*.
168BR02-Y74A	168 *lacA*::(P*xyl-tcdR-Y74A*, Erm^{R}), *amyE*::(P*tcdB-s-lacZ*, Spc^{R}). The *lacA* of 168B02 was replaced with the xylose-induced expression cassette of *tcdR-Y74A*.
168BR02-F63AI71A	168 *lacA*::(P*xyl-tcdR-F63AI71A*, Erm^{R}), *amyE*::(P*tcdB-s-lacZ*, Spc^{R}). The *lacA* of 168B02 was replaced with the xylose-induced expression cassette of *tcdR-F63AI71A*.
168BR02-I71AY74A	168 *lacA*::(P*xyl-tcdR-I71AY74A*, Erm^{R}), *amyE*::(P*tcdB-s-lacZ*, Spc^{R}). The *lacA* of 168B02 was replaced with the xylose-induced expression cassette of *tcdR-I71AY74A*.
168BR02-F63AY74A	168 *lacA*::(P*xyl-tcdR-F63AY74A*, Erm^{R}), *amyE*::(P*tcdB-s-lacZ*, Spc^{R}). The *lacA* of 168B02 was replaced with the xylose-induced expression cassette of *tcdR-F63AY74A*.

168BR02-F63AI71AY74A	168 *lacA*::(P*xyl-tcdR-F63AI71AY74A*, Erm^{R}), *amyE*::(P*tcdB-s-lacZ*, Spc^{R}). The *lacA* of 168B02 was replaced with the xylose-induced expression cassette of *tcdR-F63AI71AY74A*.

Table S3. Plasmids used in this study.

Plasmid	Description	Source
pAX01	Plasmid for gene integration and xylose-induced expression in *Bacillus subtilis*	Härtl et al. 2001, J. Bacteriol. 183, 2696-2699.
pUC19	The starting plasmid for constructing pUT series plasmids	Lab stock
pAT01	pAX01 derivative for integration and xylose-induced expression of the *tcdR* gene in *Bacillus subtilis*	All plasmids below were constructed in this study.
pUT04	pUC19 derivative for integration of the *lacZ* gene and P*tcdA* in *Bacillus subtilis*	
pUT07	pUC19 derivative for integration of the *lacZ* gene and P*tcdB* in *Bacillus subtilis*	
pUT08	pUC19 derivative for integration of the *lacZ* gene and P1*tcdR* in *Bacillus subtilis*	
pUT09	pUC19 derivative for integration of the *lacZ* gene and P2*tcdR* in *Bacillus subtilis*	
pUT11	pUC19 derivative for integration of the *lacZ* gene and P*tcdR* in *Bacillus subtilis*	
pUT12	pUT04 derivative for integration of the *lacZ* gene and P*tcdA*-s in *Bacillus subtilis*	
pUT13	pUT07 derivative for integration of the *lacZ* gene and P*tcdB*-s in *Bacillus subtilis*	
pUT14	pUT08 derivative for integration of the *lacZ* gene and P1*tcdR*-s in *Bacillus subtilis*	
pUT15	pUT09 derivative for integration of the *lacZ* gene and P2*tcdR*-s in *Bacillus subtilis*	
pUT16	pUT12 derivative for integration of the *lacZ* gene and P*tcdA*-sR1 in *Bacillus subtilis*	
pUT17	pUT14 derivative for integration of the *lacZ* gene and P1*tcdR*-sA in *Bacillus subtilis*	
pUT18	pUT12 derivative for integration of the *lacZ* gene and promoter $P_{A\text{-}1A}$ in *Bacillus subtilis*	
pUT19	pUT12 derivative for integration of the *lacZ* gene and promoter $P_{A\text{-}1T}$ in *Bacillus subtilis*	
pUT20	pUT12 derivative for integration of the *lacZ* gene and promoter $P_{A\text{-}2C}$ in *Bacillus subtilis*	
pUT21	pUT12 derivative for integration of the *lacZ* gene and promoter $P_{A\text{-}2G}$ in *Bacillus subtilis*	
pUT22	pUT12 derivative for integration of the *lacZ* gene and promoter $P_{A\text{-}3A}$ in *Bacillus subtilis*	
pUT23	pUT12 derivative for integration of the *lacZ* gene and promoter $P_{A\text{-}3T}$ in *Bacillus subtilis*	
pUT24	pUT12 derivative for integration of the *lacZ* gene and promoter $P_{A\text{-}4A}$ in *Bacillus subtilis*	
pUT25	pUT12 derivative for integration of the *lacZ* gene and promoter $P_{A\text{-}5C}$ in *Bacillus subtilis*	

pUT26	pUT14 derivative for integration of the *lac*Z gene and promoter P$_{R\text{-}1C}$ in *Bacillus subtilis*
pUT27	pUT14 derivative for integration of the *lac*Z gene and promoter P$_{R\text{-}2T}$ in *Bacillus subtilis*
pUT28	pUT14 derivative for integration of the *lac*Z gene and the mutant promoter P$_{R\text{-}3C}$ in *Bacillus subtilis*
pUT29	pUT14 derivative for integration of the *lac*Z gene and the mutant promoter P$_{R\text{-}4C}$ in *Bacillus subtilis*
pUT30	pUT14 derivative for integration of the *lac*Z gene and the mutant promoter P$_{R\text{-}5T}$ in *Bacillus subtilis*
pUT37	pUT14 derivative for integration of the *lac*Z gene and the mutant promoter P*tcdA*-sB in *Bacillus subtilis*
pUT38	pUT14 derivative for integration of the *lac*Z gene and the mutant promoter P*tcdB*-sA in *Bacillus subtilis*
pUT44	pUT14 derivative for integration of the *lac*Z gene and the mutant promoter P*tcdA*-sR2 in *Bacillus subtilis*
pAT12	pAT01 derivative for xylose-induced expression of TcdR-F63A mutant in *Bacillus subtilis*
pAT13	pAT01 derivative for xylose-induced expression of TcdR-I71A mutant in *Bacillus subtilis*
pAT14	pAT01 derivative for xylose-induced expression of TcdR-Y74A mutant in *Bacillus subtilis*
pAT15	pAT01 derivative for xylose-induced expression of TcdR-I71AY74A mutant in *Bacillus subtilis*
pAT16	pAT01 derivative for xylose-induced expression of TcdR-F63AI71AY74A mutant in *Bacillus subtilis*
pAT17	pAT01 derivative for xylose-induced expression of TcdR-F63AI71A mutant in *Bacillus subtilis*
pAT18	pAT01 derivative for xylose-induced expression of TcdR-F63AY74A mutant in *Bacillus subtilis*

Table S4. Primers used in this study. The mutation sites of TcdR-dependent promoters or the *tcdR* gene are shown in red letters.

Primer name	Sequence (5′ to 3′)	Used for
T01	GAGTGCGGCCGCCCGCGGAGCTCATGCAAAAGTCTTTTATGAATT	Construction of plasmid pAT01
T02	CAAAGGGGGAAATGGGATCCTTACAAGTTAAAATAATTTTCATAG	
T03	AATTCATAAAAAGACTTTTGCATGAGCTCCCGCGGGCGGCCGCACTC	
T04	CTATGAAAATTATTTTAACTTGTAAGGATCCCATTTCCCCCTTTG	
U01-F	CTCTTTCACGTACCCATGGCACAAAGATGGTGCATGGTCAG	Construction of plasmids for the TcdR-dependent promoters (P*tcdA*, P*tcdB*, P1*tcdR*, P2*tcdR*, P*tcdR*). pUT04
U01-R	CTTATTAGTTAATCTTTTCTCCTCTTCTATTAGATAAGGAG	pUT04
U02-F	CTCCTTATCTAATAGAAGAGGAGAAAAGATTAACTAATAAG	pUT04
U02-R	CTGACCATGCACCATCTTTGTGCCATGGGTACGTGAAAGAG	pUT04
U03-F	CTTTTCTCTTTCACGTACCCATGGTTAATGAGCTTAAAGAAATATTTAC	pUT07

U03-R	CTCCTTATTAGTTAATCTTTTCTCGCTCTTCGATTGAATAAAGACGTTG	pUT07
U04-F	GTAAATATTTCTTTAAGCTCATTAACCATGGGTACGTGAAAGAGAAAAG	pUT07
U04-R	CAACGTCTTTATTCAATCGAAGAGCGAGAAAAGATTAACTAATAAGGAG	pUT07
U05-F	CTTTTCTCTTTCACGTACCCATGGAACAAAATATTAAATAATTCTACTC	pUT08
U05-R	CTCCTTATTAGTTAATCTTTTCTCTTTTAAAATAGATCAATTTTCAAG	pUT08
U06-F	CTTGAAAATTGATCTATTTTAAAAGAGAAAAGATTAACTAATAAGGAG	pUT08
U06-R	GAGTAGAATTATTTAATATTTTGTTCCATGGGTACGTGAAAGAGAAAAG	pUT08
U07-F	CTTTTCTCTTTCACGTACCCATGGATTTTAAAATCTAGTTATAACTTC	pUT09
U07-R	CTCCTTATTAGTTAATCTTTTCTCTAAATAAAGTATTTTAGCAATAAAC	pUT09
U08-F	GTTTATTGCTAAAATACTTTATTTAGAGAAAAGATTAACTAATAAGGAG	pUT09
U08-R	GAAGTTATAACTAGATTTTAAAATCCATGGGTACGTGAAAGAGAAAAG	pUT09
U11-F	GCTTTTCTCTTTCACGTACCCATGGATTTTAAAATCTAGTTATAACTTC	pUT11
U11-R	CTCCTTATTAGTTAATCTTTTCTCATAATGATGATTTATTTGAAAATTTTG	pUT11
U12-F	CAAAATTTTCAAATAAATCATCATTATGAGAAAAGATTAACTAATAAGGAG	pUT11
U12-R	GAAGTTATAACTAGATTTTAAAATCCATGGGTACGTGAAAGAGAAAAGC	pUT11
U13-F	CTCTTTCACGTACCCATGGCATTAAAAAAATATAAGATATG	Construction of plasmids for the short version promoters (P*tcdA*-s, P*tcdB*-s, P1*tcdR*-s, P2*tcdR*-s)<br>pUT12
U13-R	CCATGGGTACGTGAAAGAG	pUT12; pUT13; pUT14
U13-2-F	CAGACAATCTCCTTATCTAATAAGAAAAGATTAACTAATAAG	pUT12
U13-2-R	TATTAGATAAGGAGATTGTCTG	pUT12
U14-F	CTCTTTCACGTACCCATGGATATTTTATATAGAACAAAG	pUT13
U14-2-F	GACAACGTCTTTATTCAATCGAGAAAAGATTAACTAATAAG	pUT13
U14-2-R	CGATTGAATAAAGACGTTGTC	pUT13
U15-F	CTCTTTCACGTACCCATGGTTATTGACTAAATTATAAAG	pUT14
U15-2-F	TAATTATGTAATTGTTACTTGAGAAAAGATTAACTAATAAG	pUT14
U15-2-R	CAAGTAACAATTACATAATTA	pUT14
U16-F	CTCTTTCACGTACCCATGGTTTAATAAAATGATTTGTTTTTAC	pUT15
U16-R	CCTTATTAGTTAATCTTTTCTTTTTAGCAATAAACTTTATAT	pUT15
U17-F	ATATAAAGTTTATTGCTAAAAAGAAAAGATTAACTAATAAGG	pUT15
U17-R	GTAAAAACAAATCATTTTATTAAACCATGGGTACGTGAAAGAG	pUT15

U18-F	CAAATTACTATCAGACAATTGTAATATCTAATAAGAAAAG ATTAAC	Construction of plasmids for P*tcdA*-s mutants. pUT16
U18-R	GTTAATCTTTTCTTATTAGATATTACAATTGTCTGATAGTAA TTTG	pUT16
U20-F	CAAATTACTATCAGACAATATCCTTATCTAATAAGAAAAG	pUT18
U20-R	CTTTTCTTATTAGATAAGGATATTGTCTGATAGTAATTTG	pUT18
U21-F	CAAATTACTATCAGACAATTTCCTTATCTAATAAGAAAAG	pUT19
U21-R	CTTTTCTTATTAGATAAGGAAATTGTCTGATAGTAATTTG	pUT19
U22-F	CAAATTACTATCAGACAATCCCCTTATCTAATAAGAAAAG	pUT20
U22-R	CTTTTCTTATTAGATAAGGGGATTGTCTGATAGTAATTTG	pUT20
U23-F	CAAATTACTATCAGACAATCGCCTTATCTAATAAGAAAAG	pUT21
U23-R	CTTTTCTTATTAGATAAGGCGATTGTCTGATAGTAATTTG	pUT21
U24-F	CAAATTACTATCAGACAATCTACTTATCTAATAAGAAAAG	pUT22
U24-R	CTTTTCTTATTAGATAAGTAGATTGTCTGATAGTAATTTG	pUT22
U25-F	CAAATTACTATCAGACAATCTTCTTATCTAATAAGAAAAG	pUT23
U25-R	CTTTTCTTATTAGATAAGAAGATTGTCTGATAGTAATTTG	pUT23
U26-F	CAAATTACTATCAGACAATCTCATTATCTAATAAGAAAAG	pUT24
U26-R	CTTTTCTTATTAGATAATGAGATTGTCTGATAGTAATTTG	pUT24
U27-F	CAAATTACTATCAGACAATCTCCCTATCTAATAAGAAAAG ATTAAC	pUT25
U27-R	GTTAATCTTTTCTTATTAGATAGGGAGATTGTCTGATAGTAA TTTG	pUT25
U19-F	CATAATTATTTAATAATTACTCCTTTGTTACTTGAGAAAAGA TTAAC	Construction of plasmids for P1*tcdR*-s mutants. pUT17
U19-R	GTTAATCTTTTCTCAAGTAACAAAGGAGTAATTATTAAATA ATTATG	pUT17
U28-F	CATAATTATTTAATAATTACGTAATTGTTACTTGAGAAAAG	pUT26
U28-R	CTTTTCTCAAGTAACAATTACGTAATTATTAAATAATTATG	pUT26
U29-F	CATAATTATTTAATAATTATTTAATTGTTACTTGAGAAAAG	pUT27
U29-R	CTTTTCTCAAGTAACAATTAAATAATTATTAAATAATTATG	pUT27
U30-F	CATAATTATTTAATAATTATGCAATTGTTACTTGAGAAAAG	pUT28
U30-R	CTTTTCTCAAGTAACAATTGCATAATTATTAAATAATTATG	pUT28
U31-F	CATAATTATTTAATAATTATGTCATTGTTACTTGAGAAAAG ATTAAC	pUT29
U31-R	GTTAATCTTTTCTCAAGTAACAATGACATAATTATTAAATA ATTATG	pUT29
U32-F	CATAATTATTTAATAATTATGTATTTGTTACTTGAGAAAAG ATTAAC	pUT30
U32-R	GTTAATCTTTTCTCAAGTAACAAATACATAATTATTAAATA ATTATG	pUT30
U37-F	CAAATTACTATCAGACAATGTCTTTATCTAATAAGAAAAGA TTAAC	pUT37
U37-R	GTTAATCTTTTCTTATTAGATAAAGACATTGTCTGATAGTAA TTTG	pUT37
U38-F	CATATTTATTTCAGACAACCTCCTTATTCAATCGAGAAAAG ATTAAC	pUT38
U38-R	GTTAATCTTTTCTCGATTGAATAAGGAGGTTGTCTGAAATA AATATG	pUT38
U44-F	CAAATTACTATCAGACAATAGTTTAATCTAATAAGAAAAG ATTAAC	pUT44

U44-R	GTTAATCTTTTCTTATTAGATTAAACTATTGTCTGATAGTAATTTG	pUT44
T05-F	GCATCGCTTTTTTCTGAAGCGCTACTTAATTTAATAT	Construction of plasmids for expression of TcdR mutants. pAT12, pAT16, pAT17, pAT18
T05-R	ATATTAAATTAAGTAGCGCTTCAGAAAAAAGCGATGC	pAT12, pAT16, pAT17, pAT18
T06-F	GATTTATGAATATATTTGACTGCAATAGCATCGCTTTTTTCTG	pAT13, pAT17
T06-R	CAGAAAAAAGCGATGCTATTGCAGTCAAATATATTCATAAATC	pAT13, pAT17
T07-F	CAGTAATGATTTATGAATAGCTTTGACTATAATAGCATCGC	pAT14, pAT18
T07-R	GCGATGCTATTATAGTCAAAGCTATTCATAAATCATTACTG	pAT14, pAT18
T08-F	CAGTAATGATTTATGAATAGCTTTGACTGCAATAGCATCGCTTTTTTCTG	pAT15, pAT16
T08-R	CAGAAAAAAGCGATGCTATTGCAGTCAAAGCTATTCATAAATCATTACTG	pAT15, pAT16
